# The neutrophil elastase inhibitor, sivelestat, attenuates acute lung injury in patients with cardiopulmonary bypass

**DOI:** 10.3389/fimmu.2023.1082830

**Published:** 2023-01-24

**Authors:** Tuo Pan, Tayierjiang Tuoerxun, Xi Chen, Cheng-Jin Yang, Chen-Yu Jiang, Yi-Fan Zhu, Ze-Shi Li, Xin-Yi Jiang, Hai-Tao Zhang, He Zhang, Ya-Peng Wang, Wei Chen, Li-Chong Lu, Min Ge, Yong-Qing Cheng, Dong-Jin Wang, Qing Zhou

**Affiliations:** ^1^ Department of Cardio-Thoracic Surgery, Nanjing Drum Tower Hospital, Affiliated Drum Tower Hospital, Medical School of Nanjing University, Nanjing, China; ^2^ Department of Cardio-Thoracic Surgery, Nanjing Drum Tower Hospital, Peking Union Medical College & Chinese Academy of Medical Sciences, Graduate School of Peking Union Medical College, Nanjing, China; ^3^ The Jockey Club School of Public Health and Primary Care, The Chinese University of Hong Kong, Hong Kong, Hong Kong SAR, China; ^4^ Department of Pediatric Surgery, Sanya Women and Children’s Hospital, Sanya, China; ^5^ Department of Cardio-Thoracic Surgery, Shanghai Children’s Medical Center, School of Medicine, Shanghai Jiao Tong University, Shanghai, China

**Keywords:** sivelestat, cardiopulmonary bypass, acute lung injury, cardiovascular surgery, outcomes

## Abstract

**Background:**

The sivelestat is a neutrophil elastase inhibitor thought to have an effect against acute lung injury (ALI) in patients after scheduled cardiac surgery. However, the beneficial effect of sivelestat in patients undergoing emergent cardiovascular surgery remains unclear. We aim to evaluate the effect of sivelestat on pulmonary protection in patients with ALI after emergent cardiovascular surgery.

**Methods:**

Firstly, a case-control study in 665 patients undergoing emergent cardiovascular surgery from January 1^st^, 2020 to October 26^th^, 2022 was performed. 52 patients who received sivelestat (0.2mg/kg/h for 3 days) and 613 age- and sex-matched controls. Secondly, a propensity-score matched cohort (sivelestat vs control: 50 vs 50) was performed in these 665 patients. The primary outcome was a composite of adverse outcomes, including 30-day mortality, ECMO, continuous renal replacement therapy (CRRT) and IABP, etc. The secondary outcome included pneumonia, ventricular arrhythmias and mechanical ventilation time, etc.

**Results:**

In propensity-matched patients, the 30-day mortality (16% vs 24%, P=0.32), stroke (2% vs 8%, P=0.17), ECMO(6% vs 10%, P=0.46), IABP(4% vs 8%, P=0.40) and CRRT(8% vs 20%, P=0.08) had no differences between sivelestat and control group; sivelestat could significantly decrease pneumonia (40% vs 62%, P=0.03), mechanical ventilation time (median: 96hours, IQR:72-120hours vs median:148hours, IQR:110-186hours, P<0.01), bilateral pulmonary infiltrates (P<0.01), oxygen index (P<0.01), interleukin-6(P=0.02), procalcitonin(P<0.01) and C-reactive protein(P<0.01).

**Conclusion:**

Administration of sivelestat might improve postoperative outcomes in patients with ALI after emergent cardiovascular surgery. Our results show that sivelestat may be considered to protect pulmonary function against inflammatory injury by CPB.

**Registration:**

http://www.chictr.org.cn/showproj.aspx?proj=166643, identifier ChiCTR2200059102.

## Introduction

Cardiopulmonary bypass (CPB) is a necessary life support during open-heart surgery. Systemic inflammatory response syndrome (SIRS) caused by CPB has been well known to increase postoperative morbidity and mortality (1, 2). Acute respiratory distress syndrome (ARDS) and acute lung injury (ALI), which are characterized by pulmonary edema associated with SIRS, are also induced after CPB and significantly contribute to postoperative morbidity and mortality (3–6).

Documented components of the inflammatory reaction include the activation of complement, increased surface expression of adhesion molecules on leukocytes, and the presence of pro-inflammatory cytokines in the systemic circulation (7–12). Neutrophils which is a main part of leukocytes plays an important role in SIRS through the production of superoxide radicals and the release of chemical mediators (12, 13). It has been proven that activated neutrophils is one of the most important initiating events of pulmonary dysfunction induced by CPB (14).

Sivelestat is a synthetic, specific, low molecular-weight neutrophil elastase inhibitor (15). It has been shown to reduce both neutrophil elastase levels and interleukin-6 production and to preserve neutrophil deformability during extracorporeal circulation (6, 16, 17). Several clinical studies have shown the beneficial effect of sivelestat for patients undergoing cardiovascular surgery with CPB (6, 12). However, these studies evaluated only the scheduled cardiac surgery. The emergent cardiovascular surgery usually had more severe ALI compared with scheduled cardiac surgery (15, 18). This agent may prevent the adverse reaction of SIRS and could be one of the best therapies to attenuate ALI in patients undergoing emergent cardiovascular surgery. We, therefore, designed this study to evaluate the effect of sivelestat on pulmonary protection in patients with ALI after emergent cardiovascular surgery.

## Materials and methods

### Study population

This study is an investigator-initiated cohort study. It was initiated on January 1^st^, 2020, and completed on October 26^th^, 2022. This study was approval by the ethical committee of Nanjing Drum Tower Hospital (2022-102-01) and registered in the Chinese Clinical Trial Registry (ChiCTR2200059102). The study enrolled 665 patients with CPB from the department of cardiothoracic in Nanjing Drum Tower Hospital. Before enrollment, all patients had signed informed consent forms.

The inclusion criteria were as follows: adult patients undergoing on-pump emergent cardiovascular surgery; aged > 18 years old, oxygen partial pressure/fraction of inspired O_2_ (PaO_2_/FiO_2_) <150mmHg at the end of CPB.

The exclusion criteria were as follows: patients undergoing non-emergent surgery; deep hypothermic circulatory arrest (DHCA) was more than 25 minutes; poor hepatorenal dysfunction (19) (Child-Pugh Class B or C, estimated glomerular filtration rate <35 mL/min/1.73m^2^); cardiogenic shock at the end of CPB (20) (vasoactive inotropic score>40, cardiac index <2.2L/min·m^2^, mean arterial pressure <65mmHg); fluid overload at the end of CPB (21) (inferior vena cava > 21mm); baseline inflammatory indicators abnormal (19) (interleukin-6 (IL-6) >10pg/mL, procalcitonin (PCT) >0.5 ng/mL, C reactive protein (CRP) >10 mg/L); diagnosed with inflammatory immune diseases, infectious or tumor disease; received treatment of sivelestat previously; had sivelestat allergy or intolerance; pregnancy.

Based on the retrospective review of our institution’s database, 816 patients met the inclusion and exclusion criteria in our study. The sivelestat was routinely used in our hospital on the 1^st^ January 2022. Therefore, 52 patients who received sivelestat were assigned to the sivelestat group. To investigate whether sivelestat could improve outcomes, we selected 613 matched controls from the remaining 764 patients (control group). The control subjects were selected for each case and matched for sex and age (± 2 years).

### Medical intervention

This section was available in the online supplement.

### Definition and data collection

The follow-up ended on October 26^th^, 2022. In the matched cohort, patients were followed for 26.3 ± 7.6(median: 30, IQR: 28–30) days, and no patients were lost at follow-up. The primary outcome was a composite of adverse outcomes, including 30-day mortality, ECMO, continuous renal replacement therapy (CRRT) and IABP, etc. The secondary outcome included pneumonia, ventricular arrhythmias and mechanical ventilation time, etc. The detailed variables had been shown in **Table 1**. The vasoactive inotropic score (VIS) (22) was as follows: dobutamine dose (μg/kg/min) + dopamine dose (μg/kg/min) + [10,000×vasopressin dose (U/kg/min)] + [100×norepinephrine dose (μg/kg/min)].

**Table 1:** Postoperative outcomes in propensity-matched patients.

Variable	Sivelestat (n=50)	Control (n=50)	P value
Primary outcomes (n, %)	8,16%	12,24%	0.32
30-day mortality	8,16%	12,24%	0.32
ECMO use	3,6%	5,10%	0.46
IABP use	2,4%	4,8%	0.40
Stroke	1,2%	4,8%	0.17
CRRT use	4,8%	10,20%	0.08
Secondary outcomes (n, %)			
Pneumonia	20,40%	31,62%	0.03
Ventricular arrhythmias	4,8%	10,20%	0.08
High arterial lactic > 48 hours	20,40%	35,70%	0.003
New-onset atrial fibrillation	2,4%	8,16%	0.05
Length of ICU stay (days)	7.0(6.0-9.2)	9.0(8.0-14.2)	<0.01^*^
MV time (hours)	96(72-120)	148(110-186)	<0.01^*^
BPI on POD2 (n, %)			<0.01^*^
Mild	29,58%	14,28%	
Moderate	16,32%	25,50%	
Severe	5,10%	11,22%	

MV, Mechanical ventilation; CRRT, Continuous renal replacement therapy; BPI, Bilateral pulmonary infiltrates on chest X ray; Median (Interquartile range); ECMO, Extracorporeal membrane oxygenation; IABP, Intra-aortic ballon pump; High arterial lactic, arterial lactic > 2 mmol/L; POD2, The second postoperative day.

*P<0.0001.

### Statistical analysis

The SPSS statistical software (version 24) was used for analysis. The mean ± SD or median (interquartile range) were used to present continuous variables. Discrete variables are depicted as frequencies (n, %). The Student’s t-test was used to evaluate normally distributed continuous variables. The Mann-Whitney U nonparametric method was used for non-normally distributed continuous variables. Continuous variables were determined to be normally distributed by the Shapiro-Wilk test. The chi-Square test or Fisher’s exact test was used to compare categorical data. Differences between the two groups were also analyzed by repeated measures ANOVA considering the repeated measurements from POD1 to POD5. R software (version:4.2.2) was used for univariate analysis. In univariate analysis, the study cohort (n=665) was divided into the adverse events group and the control group. The adverse events include 30-day mortality, ECMO, CRRT, IABP, and stroke. It is the same as the definition of primary outcomes. Covariates reaching statistical significance (P ≤ 0.10) in the univariate analysis were entered into a forward selection multivariable logistic regression model. Collinearity diagnostics were performed using tolerance estimates for individual variables in a linear regression model.

The possibility of the existence of bias may affect our findings. To avoid bias, we used a propensity score to adjust the study cohort. This methodology permitted the comparison of patients who received sivelestat against control with a similar risk profile. Propensity score 1-to-1 matching was utilized with the nearest neighbor algorithm without replacement and a 0.01 caliper set (23). Age, gender, body mass index (BMI), left ventricular ejection fraction (LVEF), type of cardiac surgery, CPB, DHCA, and diabetes were put into a logistic regression model to estimate the propensity score. The standardized mean differences (absolute SMD < 10%) are used to assess pre-match imbalances and post-match balance. A P value of < 0.05 was considered statistically significant.

## Results

**Figure 1:**
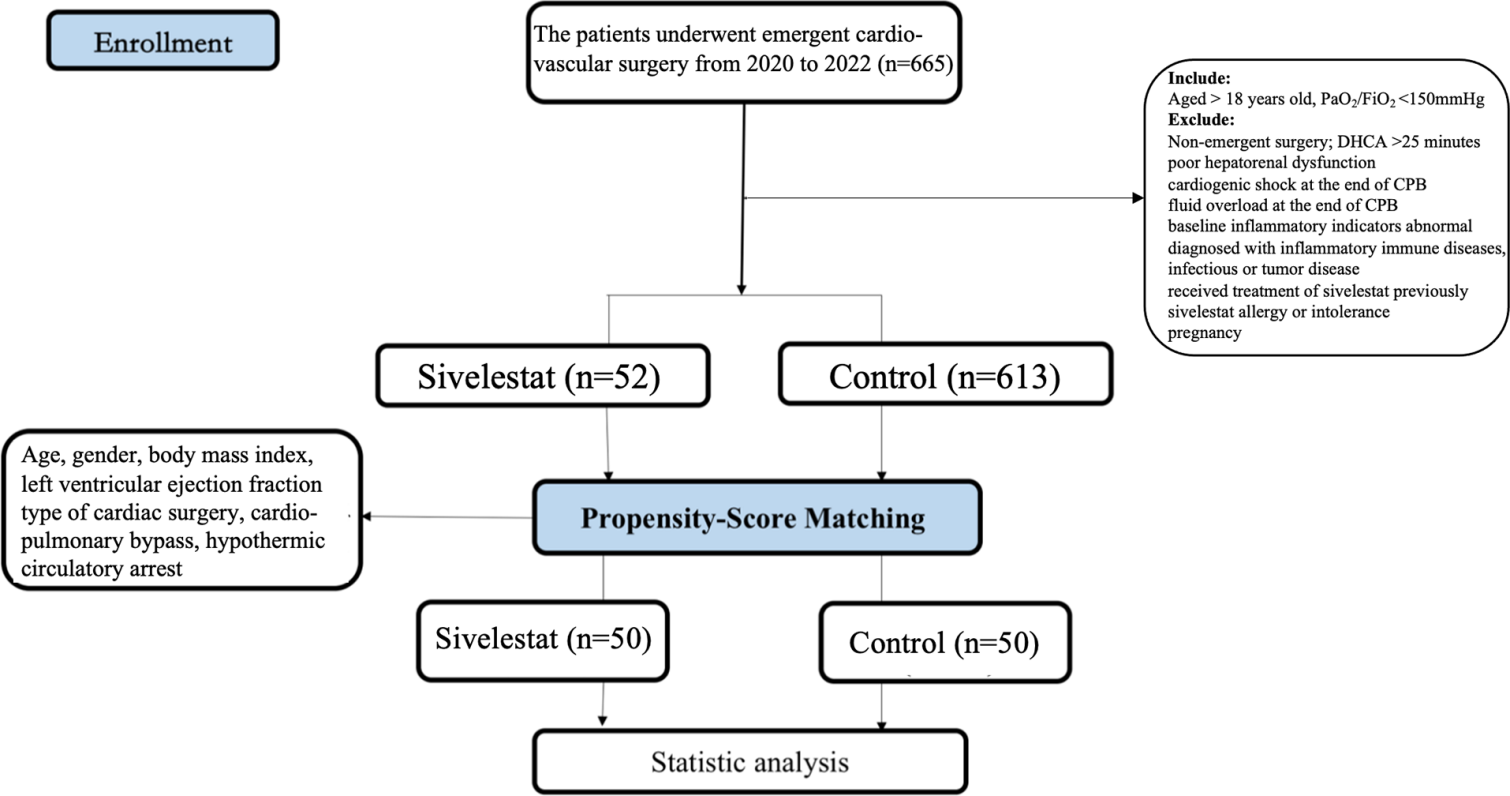
Enrollment flow chart.

After propensity matching, a total of 100 patients were enrolled in our study (**Figure 1**, 50 in the sivelestat group and 50 in the control group). Before and after propensity-score matching, the SMD of age, gender, BMI, LVEF, type of cardiac surgery, CPB, DHCA, and diabetes were shown in **eFigure 1** (online supplementary). The absolute SMDs of these variables were <10%. Therefore, these variables were well balanced after propensity matching.

**Figure 2:**
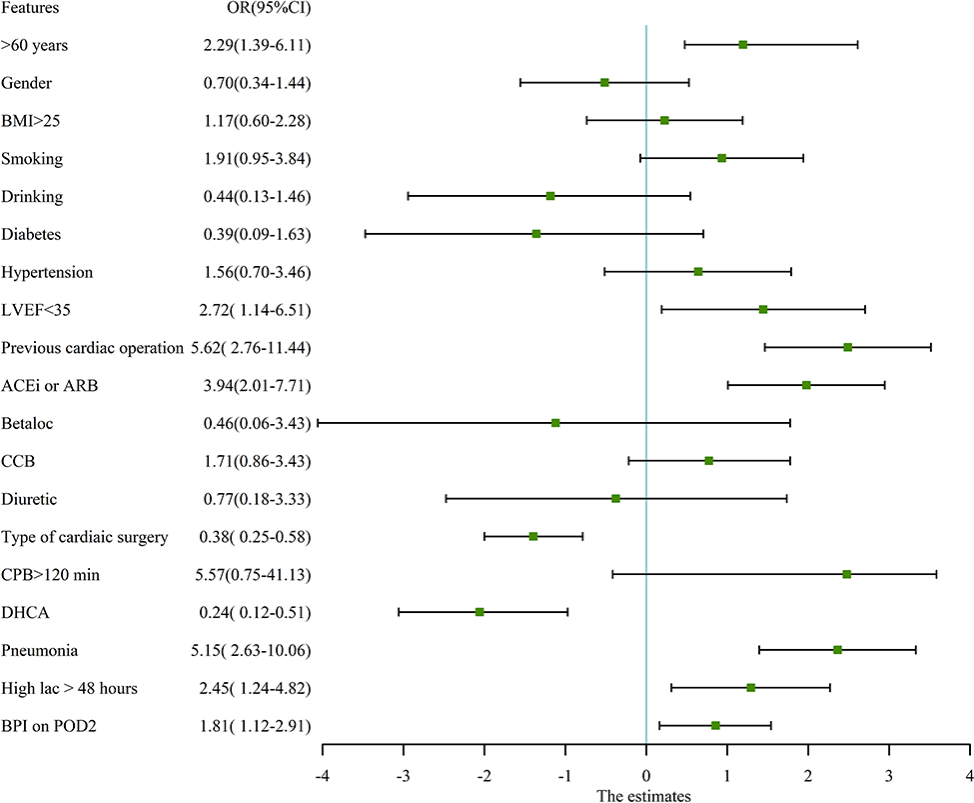
univariate analysis for adverse events in pre-propensity matched patients. The figure applied log2 transformation in order to make it easy to read. BMI, Body mass index; LVEF, Left ventricular ejection fraction; ACEi, Angiotensin-converting enzyme inhibitors; ARB, Angiotensin receptor blockers; CCB, Calcium channel blocker; CPB, Cardiopulmonary bypass; DHCA, Deep hypothermic circulation arrest; Betaloc, β-blocker; BPI, Bilateral pulmonary infiltrates on chest X ray.

In our study cohort, the baseline and demographic variables were not different between the two groups (**Table 2**). There were no gastrointestinal disturbances observed in the two groups. There were 65 patients who underwent emergent on-pump coronary artery bypass grafting (CABG), 383 patients who underwent aortic arch replacement because of aortic dissection, 26 patients who underwent emergent left atrial myxoma resection, 4 patients who underwent emergent repair of ruptured sinus of valsalva aneurysm, and 187 patients who underwent emergent valvular replacement because of acute heart failure or left atrial thrombus. Before propensity matching, adverse events occurred in 38 patients (5.71%), including 30-day mortality (n=20), ECMO (n=11), CRRT (n=25), IABP (n=18), and stroke (n=9). The odds ratio (OR) and 95% confidence interval (CI) had large variations. In univariate analysis, age > 60 years-old (OR:2.29, 95%CI:1.39-6.11, P<0.01), pre-operative LVEF<35% (OR:2.72, 95%CI: 1.14-6.51, P=0.02), previous cardiac operation (OR: 5.62, 95%CI: 2.757-11.44, P<0.01), pre-operative ACEi/ARB (OR: 3.94, 95%CI:2.01-7.71, P<0.01), type of cardiac surgery (OR: 0.38, 95%CI: 0.25-0.58, P<0.01), CPB time>2 hours (OR:5.57, 95%CI:0.75-41.13, P=0.09), DHCA (OR:0.24, 95%CI: 0.12-0.51, P<0.01), postoperative pneumonia (OR: 5.15, 95%CI: 2.63-10.06, P<0.01), high arterial lactic > 48 hours (OR: 2.45, 95%CI: 1.24-4.82, P=0.01), and bilateral pulmonary infiltrates (BPI) on chest X- ray on the POD2 (OR: 1.81, 95%CI: 1.12-2.91, P=0.014) decreased or increased the rate of adverse events. The detailed variables of univariate analysis have been shown in **Figure 2** which applied log2 transformation.

**Table 2:** Characteristics in pre- and after- propensity matching.

Variables	Pre-Propensity Matched			After-Propensity Matched		
	Sivelestat (N=52)	Control (n=613)	P value	Sivelestat (n=50)	Control (n=50)	P value
Age (year)	58.5±14.2	58.9±13.7	0.84	56.2±12.9	56.1±13.5	0.95
Gender (n,%)^*^	37,71.1%	387,63.1%	0.29	37,74%	34,68%	0.51
BMI (kg/m^2^)	25.0 (23.0-28.4)	23.7 (22.4-26.1)	0.004	24.9±3.1	24.4±2.5	0.37
LVEF (%)	53.3±9.1	51.5±9.3	0.32	53.1±8.3	53.8±6.1	0.81
History (n,%)						
Diabetes	7,13.5%	74,12.1%	0.82	7,14%	6,12%	0.77
Hypertension	37,71.1%	436,71.1%	0.99	36,72%	34,68%	0.66
CKD	0	0	—	0	0	—
CLD	0	0	—	0	0	—
Stroke	0	0	—	0	0	—
Marfan	0	2,0.3%	0.68	0	0	—
Liver disease	0	6,1.0%	0.47	0	0	—
Smoking	13,25%	134,21.6%	0.60	13,26%	9,18%	0.33
Drinking	9,17.3%	96,15.7%	0.75	6,12%	4,8%	0.51
PCO	6,11.5%	67,19.9%	0.89	5,10%	4,8%	0.73
AF	6,11.5%	74,12.1%	0.91	5,10%	4,8%	0.73
β-blocker	3,5.8%	33,5.4%	0.75	3,6%	2,4%	0.65
ACEi/ARB	10,19.2%	114,18.6%	0.85	8,16%	6,12%	0.56
CCB	12,23.1%	147,24.0%	0.88	11,22%	18,36%	0.12
Digitalis	0	0	—	0	0	—
Amiodarone	0	0	—	0	0	—
Diuretic	4,7.7%	40,6.5%	0.74	2,4%	2,4%	—
Clopidogrel	1,1.9%	10,1.6%	0.87	0	0	—
Ticagrelor	0	0	—	0	0	—
Warfarin	2,3.8%	22,3.6%	0.92	2,4%	2,4%	—
TCO(n,%)			0.76			0.90
CABG	5,9.6%	60,9.8%		6,12%	6,12%	
VR	14,26.9%	173,28.2%		11,22%	11,22%	
AAR	30,57.7%	353,57.6%		27,54%	28,56%	
LAM resection	2,3.9%	24,3.9%		6,12%	5,10%	
SVA repair	1,1.9%	3,0.5%		0	0	
CPB (min)	181.9±46.7	183.8±48.9	0.78	180.3±48.2	174.2±54.4	0.56
DHCA (n,%)	30,57.7%	353,57.6%	0.98	27,54%	28,56%	0.84

*male.

BMI, Body mass index; LVEF, Left ventricular ejection fraction; CKD, Chronic kidney disease; CLD, Chronic lung disease; AF, Atrial fibrillation; PCO, Previous cardiac operation; ACEi, Angiotensin-converting enzyme inhibitors; ARB, Angiotensin receptor blockers; CCB, Calcium channel blocker; TCO, Type of cardiac operation; CABG, Coronary artery bypass grafting; VR, Valvular replacement; AAR, Aortic arch replacement ; SVA, Sinus of valsalva aneurysm; LAM, Left atrial myxoma; CPB, Cardiopulmonary bypass; DHCA, Deep hypothermic circulation arrest.

In multivariate regression analysis (**Table 3**), the pre-operative LVEF<35% (P=0.01), pre-operative ACEi/ARB (P=0.03), postoperative pneumonia (P<0.01), and BPI (P=0.03) on chest X-ray showed a significant difference between adverse events group and control group. After propensity matching, the mean ( ± SD) ages of the patients among the two groups were 56.21 ± 12.92 vs 56.12 ± 13.52 year-old (P=0.95), and 74% vs 68% were males (P=0.51). Body mass index (P=0.37), CPB(P=0.56), type of cardiac surgery (P=0.90), and DHCA(P=0.84) had no difference between the sivelestat group and the control group.

**Table 3:** Multivariable logistic regression in pre-propensity matched patients.

Variables	Odds ratio	95% CI	P value
Age > 60 years-old	0.73	0.25-2.14	0.57
Pre-operative LVEF<35%	3.92	1.42-10.77	0.01
Previous cardiac operation	2.70	0.98-7.40	0.06
Pre-operative ACEi/ARB	2.88	1.10-7.57	0.03
Type of cardiac surgery	0.51	0.24-1.07	0.07
CPB time>2 hours	7.08	0.87-57.59	0.06
DHCA	0.48	0.12-2.02	0.32
Postoperative pneumonia	9.38	2.79-31.55	<0.01^*^
High arterial lactic > 48 hours	0.45	0.13-1.55	0.21
BPI on the POD2	1.86	1.06-3.28	0.03

LVEF, Left ventricular ejection fraction; ACEi, Angiotensin-converting enzyme inhibitors; ARB, Angiotensin receptor blockers; CPB, Cardiopulmonary bypass; DHCA, Deep hypothermic circulation arrest; Bilateral pulmonary infiltrates on chest X ray; POD2, The second postoperative day.

High arterial lactic: arterial lactic > 2 mmol/L.

*P<0.0001.

**Figure 3:**
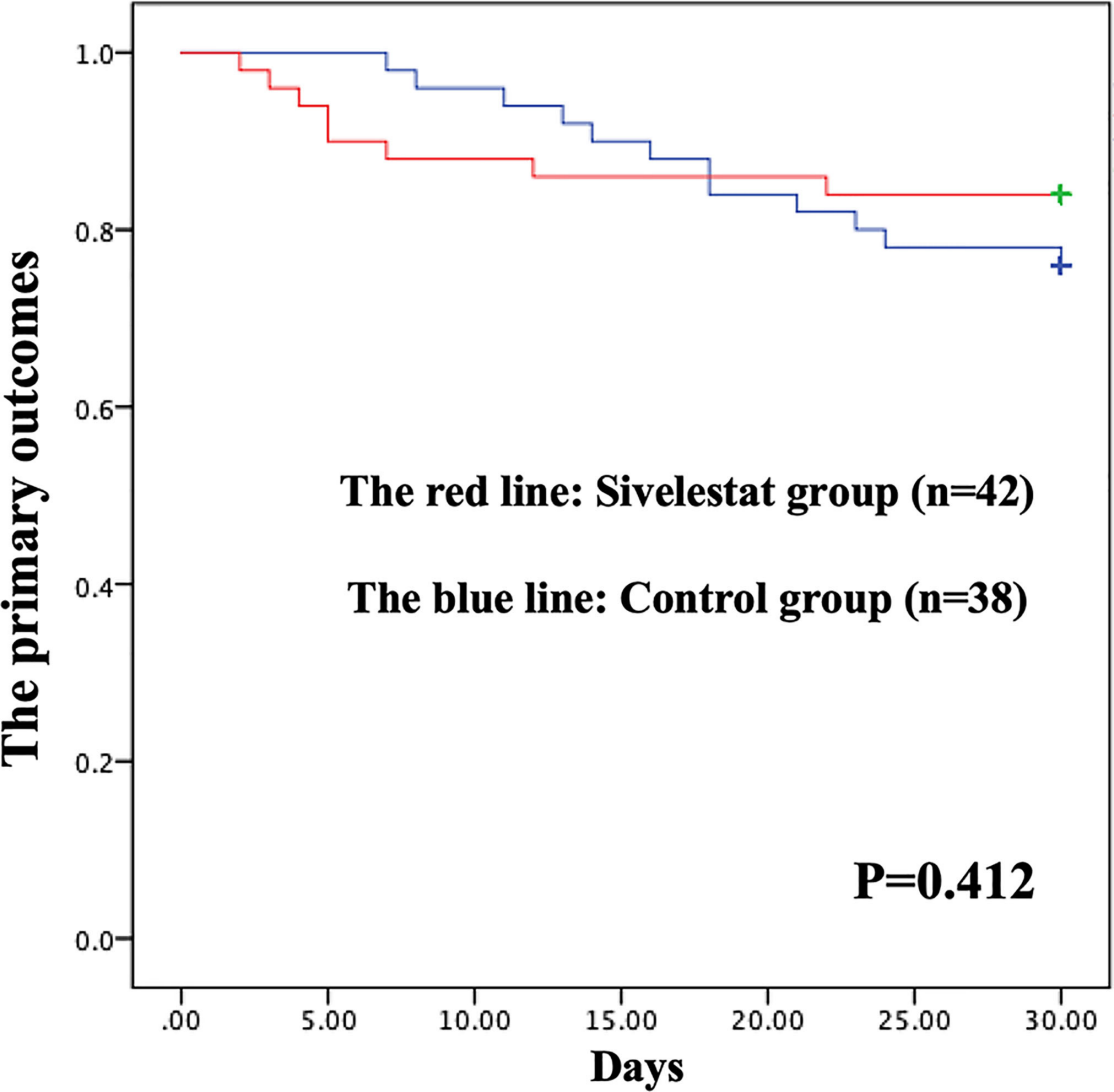
Kaplan-Meier **(K-M)** curves showing the primary outcomes had no difference between the two groups in propensity-matched patients during 30-days follow-up.

The postoperative outcomes of propensity-matched patients were presented in **Table 1**. After propensity matching, no primary outcomes (**Figure 3**), including 30-day mortality (P=0.32), ECMO (P=0.46), IABP(P=0.40), and stroke (P=0.17), had significant differences in the two groups during the 30-day follow-up. The secondary outcomes, including pneumonia (P=0.03), mechanical ventilation time (P<0.01), length of ICU (P<0.01), duration of high arterial lactate (>2mmol/L) more than 48 hours (P<0.01), showed significant differences between sivelestat and control group. Additionally, sivelestat could significantly improve the BPI on chest X-ray (P<0.01) among the two groups.

**Figure 4:**
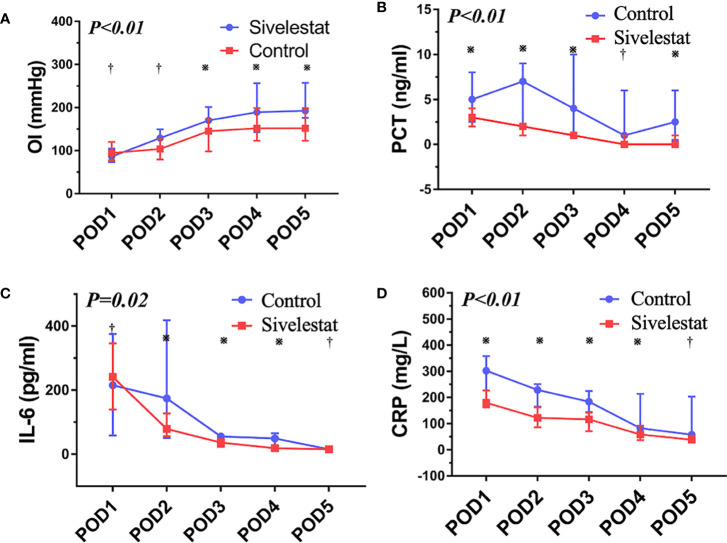
**(A)** The oxygen index (PaO_2_/FiO_2_), P < 0.01; ※:P < 0.05, ^†^:P > 0.05. **(B)** The procalcitonin, P < 0.01; ※:P < 0.05, ^†^:P > 0.05. **(C)** The interleukin-6, P=0.02; ※:P < 0.05, ^†^:P > 0.05. **(D)** The C reactive protein, P < 0.01; ※:P < 0.05, ^†^:P > 0.05.

The baseline (admission to ICU) of oxygen index (PaO_2_/FiO_2_) was a well balance between the two groups (**Table 4**). The postoperative inflammatory biomarkers, including WBC count, neutrophil, CRP, IL-6, and PCT, were significantly different between the sivelestat and control group (P<0.05). Compared with the control group, the sivelestat group showed a significantly decreased level of IL-6 (P<0.01), CRP(P<0.01) and PCT (P<0.01) on the POD3. Moreover, the sivelestat group also had lower levels of CRP, PCT, and IL-6 on POD2 (P<0.05). When analyzing with repeated measures ANOVA test to take repeated measurements into account, the sivelestat group still had a lower level of PaO_2_/FiO_2_ (**Figure 4A**, P<0.01), PCT (**Figure 4B**, P<0.01), IL-6 (**Figure 4C**, P=0.02) and CRP (**Figure 4D**, P<0.01), and had a non-significantly lower level of WBC(P=0.98) and Neutrophil (P=0.61) within postoperative 5 days compare with the control group. The detailed data could be acquired in **Table 4**.

**Table 4:** PaO2/FiO2 and biomarkers of inflammation within postoperative 5 days.

Variable	Sivelestat (n=50)	Control (n=50)	P value
Lowest PaO_2_/FiO_2_ (mmHg)			<0.01^*^
Admission to ICU	93.0 (86.1-97.2)	94.0 (86.7-104.2)	0.12
** **POD 1	94.5 (77.5-120.0)	86.0 (73.0-104.5)	0.44
** **POD 2	129.0 (107.7-149.6)	103.9 (79.6-126.7)	<0.01^*^
** **POD 3	170.2 (145.5-201.8)	145.0 (98.3-173.4)	<0.01^*^
** **POD 4	189.2 (142.9-256.5)	151.8 (122.7-198.7)	<0.01^*^
** **POD 5	192.5 (176.0-257.0)	171.5 (153.2-195.5)	<0.01^*^
White cell count (×10^9^/L)			0.37
** **POD 1	11.3 (8.0-13.6)	10.1 (8.2-12.7)	0.98
** **POD 2	11.7 (10.4-14.9)	13.1 (11.4-16.6)	0.11
** **POD 3	11.3 (9.0-11.9)	11.6 (9.9-16.0)	0.15
** **POD 4	8.6 (6.2-9.4)	8.9 (8.4-10.9)	0.06
** **POD 5	8.2 (6.9-9.4)	8.2 (7.4-10.2)	0.21
Neutrophil (%)			0.61
** **POD 1	87.1 (85.2-89.1)	88.1 (85.1-91.9)	0.24
** **POD 2	86.7 (82.4-89.4)	87.1 (82.9-89.2)	0.60
** **POD 3	87.0 (80.0-89.6)	86.1 (84.1-89.1)	0.98
** **POD 4	84.5 (81.2-86.6)	84.3 (78.5-86.2)	0.37
** **POD 5	77.5 (76.3-82.9)	77.5 (73.9-83.4)	0.50
C-reactive protein (mg/L)			<0.01^*^
** **POD 1	179.4 (162.5-226.3)	302.2 (162.7-357.5)	<0.01^*^
** **POD 2	121.9 (86.0-161.6)	228.5 (164.9-250.9)	<0.01^*^
** **POD 3	116.1 (70.5-141.9)	184.3 (143.7-224.1)	<0.01^*^
** **POD 4	58.2 (36.7-92.3)	82.3 (69.5-213.9)	<0.01^*^
** **POD 5	38.5 (34.7-53.8)	58.2 (36.3-202.5)	0.12
Interleukin-6 (pg/ml)			0.02
** **POD 1	214.6 (57.6-375.1)	240.5 (139.1-346.6)	0.36
** **POD 2	78.5 (55.7-127.4)	174.1 (50.4-418.5)	0.02
** **POD 3	36.3 (22.6-46.6)	55.2 (43.9-61.4)	<0.01^*^
** **POD 4	18.7 (13.5-27.1)	49.5 (14.5-65.1)	0.01
** **POD 5	15.1 (10.3-18.1)	15.2 (13.4-20.8)	0.27
Procalcitonin (ng/ml)			<0.01^*^
** **POD 1	2.9 (1.9-3.7)	4.7 (2.4-7.6)	0.02
** **POD 2	1.8 (0.6-2.5)	7.1 (1.5-9.4)	<0.01^*^
** **POD 3	1.1 (0.6-1.3)	3.5 (0.6-10.1)	<0.01^*^
** **POD 4	0.5 (0.2-0.8)	1.2 (0.2-5.9)	0.25
** **POD 5	0.3 (0.2-0.7)	2.4 (0.6-6.1)	<0.01^*^

POD1, The first postoperative day; POD2, The second postoperative day; POD3, The third postoperative day; POD4, The fourth postoperative day; POD5, The fifth postoperative day; ESR, Erythrocyte sedimentation rate.

*P<0.0001.

## Discussion

Acute lung injury after CPB, which is one of the most serious inflammatory reactions induced by CPB, has significantly increased morbidity and mortality (3–5). Neutrophil activation is an important initiating event of this phenomenon (14). An imbalance between neutrophil elastase and its endogenous protease inhibitors has been considered to be a possible mechanism by which neutrophil elastase causes lung tissue destruction (14). The sivelestat which is a neutrophil elastase inhibitor has been proven to be useful for anti-ALI in scheduled cardiac surgery (6, 12). However, it is still unknown what is the effect of sivelestat on emergent cardiovascular surgery. Our study showed that the postoperative adverse outcomes were acceptable (5.71%) in patients who underwent emergent cardiovascular surgery; the pre-operative LVEF<35%, postoperative pneumonia, and the BPI on chest X-ray were independent risk factors for postoperative adverse outcomes; the administration of sivelestat might improve postoperative outcomes in patients with ALI after emergent cardiovascular surgery. According to our findings, sivelestat may be considered to protect pulmonary function against inflammatory injury by CPB.

Activated neutrophils adhere to pulmonary vascular endothelial cells by expression of adhesion molecules and damage endothelial cells (5, 7–9). Moreover, activated neutrophils stimulate the production of elastase, superoxide, and cytokines (5, 7–9, 11). It has resulted in hyperpermeability of pulmonary capillaries and interstitial edema of the lung (13, 14). The ALI/ARDS are serious complications after open heart surgery. They could lead to 15% to 28% mortality in patients with CPB. Therefore, an agent which could inhibit neutrophil or neutrophil elastase may be useful and helpful to prevent ALI-related poor outcomes. Several previous studies have reported that the neutrophil elastase inhibitor, sivelestat, could significantly improve postoperative outcomes in patients with CPB (6, 12, 15, 18). However, their study populations contained congenital heart patients (6, 12) or a small sample (only enrolled 14 patients) (18). Morimoto et al. reported (15) that prophylactic administration of sivelestat at the initiation of CPB results in better postoperative pulmonary function, leading to earlier extubation time in patients who underwent total arch replacement. However, they excluded patients who underwent emergent surgery. It is well known that emergent operation has more serious SIRS compared with scheduled surgery. Our work found that sivelestat could improve the outcomes in ALI patients after CPB. It is filled a gap in emergent cardiac surgery.

The PaO_2_/FiO_2_ ratio is well established as a parameter that quantifies impaired respiratory function (24). The moderate ALI/ARDS was defined by PaO_2_/FiO_2_ ratio ≤ 200mmHg and > 100mmHg; the severe ALI/ARDS was defined by PaO_2_/FiO_2_ ratio ≤ 100mmHg (24). Our study cohort enrolled the patients who had PaO_2_/FiO_2_ ratio < 150mmHg at the end of CPB. It could ensure that the patients with ALI after CPB were enrolled in the study population. And patients with fluid overload and cardiogenic shock were excluded. Meanwhile, we used methods, including case-control and propensity score, to ensure the maximized homogeneity between the two groups. Furthermore, in our study, the inflammatory biomarkers (PCT, CRP, and IL-6) were decreased in the sivelestat group. It strengthened the evidence for the conclusion that anti-neutrophil therapies could reduce the severity of ALI. Finally, we found that WBC count and neutrophil had no difference among the two groups. The sivelestat is an inhibitor of neutrophil elastase. It does not work on the absolute value of leukocytes or neutrophils. It may be the reason why no differences in WBC count and neutrophil among the two groups.

Liu et al. reported that patients with acute type A aortic dissection (ATAAD) usually had severe inflammatory reactions, which resulted in poor short-term outcomes (25). Thourani et al. reported that emergent status on mitral valve replacement surgery and coronary artery bypass grafting (CABG) significantly increased morbidity and mortality (26). Because emergent cardiovascular surgery easily leads to severe inflammation (27). Sivelestat, which is a potential anti-inflammatory drug, may have positive effects on organ protection in patients undergoing emergent cardiovascular surgery. Our study added evidence to this hypothesis. However, our finding, which needs to be confirmed in a larger sample random control trial to assess clinical endpoints, suggests a potential role of sivelestat in the alleviation of postoperative inflammation.

In conclusion, the administration of sivelestat could improve postoperative outcomes in patients with ALI after emergent cardiovascular surgery. Our results show that sivelestat may be considered to protect pulmonary function against inflammatory injury by CPB.

## Study limitation

Our study design involves a single center’s experiences with the inherent disadvantages of a retrospective study, which is highly prone to bias. This observational study could be influenced by potential biases. We used propensity score matching to avoid these biases. However, factors that affect assignment to treatment and outcomes but that cannot be observed cannot be accounted for in the matching procedure. Any hidden bias due to latent variables might remain after matching, which could lead to some statistical faults. Moreover, with this analysis, we remove a large number of patients from the analysis but may have elevated statistical errors. Thus, the implementation of the prognostic value of sivelestat should be assessed in future studies. Furthermore, to our best knowledge, no published clinical trials reported positive or negative effects on gastrointestinal function. Gastrointestinal disturbances were also not observed in our study. It may need to confirm in the next prospective clinical trial. Furthermore, the pre-operative lung function is necessary to submit. However, lung function is not routinely implemented in our hospital. The lack of lung-function data may lead to some errors. Finally, our study found that pre-operative ACEi/ARB was an independent risk factor for postoperative adverse outcomes. ACEi/ARB are well-known drugs that could result in the greatest mortality reduction in patients with heart failure (28). Patients with heart failure with reduced ejection fraction were usually suggested to administrate ACEi/ARB (28). The pre-operative LVEF<35% was also proven to be an independent risk factor for postoperative adverse outcomes in our study. In another word, ACEi/ARB might be a confounding factor. A high-quality clinical trial might be needed to be implemented if we wanted to conclude the negative effect of ACEi/ARB on patients who underwent emergent cardiovascular surgery. These limitations of this assessment are important and should be acknowledged.

## Data availability statement

The raw data supporting the conclusions of this article will be made available by the authors, without undue reservation.

## Ethics statement

The studies involving human participants were reviewed and approved by the ethical committee of Nanjing Drum Tower Hospital. The patients/participants provided their written informed consent to participate in this study. Written informed consent was obtained from the individual(s) for the publication of any potentially identifiable images or data included in this article.

## Author contributions

TP, TT, and XC carried out the study, participated in the statistical analysis, and drafted the manuscript. C-YJ and Y-FZ participated in the design of the study. Z-SL and X-YJ participated in the sequence alignment. H-TZ and HZ performed the statistical analysis. Y-PW, WC, L-CL, and MG participated in the data collection. Y-QC, D-JW, and QZ conceived the study, participated in its design and coordination, and helped to draft the manuscript. All authors contributed to the article and approved the submitted version.
