# Corrigendum: The neutrophil elastase inhibitor, Sivelestat, attenuates acute lung injury in patients with cardiopulmonary bypass

**DOI:** 10.3389/fimmu.2023.1165081

**Published:** 2023-03-01

**Authors:** Tuo Pan, Tayierjiang Tuoerxun, Xi Chen, Cheng-Jin Yang, Chen-Yu Jiang, Yi-Fan Zhu, Ze-Shi Li, Xin-Yi Jiang, Hai-Tao Zhang, He Zhang, Ya-Peng Wang, Wei Chen, Li-Chong Lu, Min Ge, Yong-Qing Cheng, Dong-Jin Wang, Qing Zhou

**Affiliations:** ^1^ Department of Cardio-Thoracic Surgery, Nanjing Drum Tower Hospital, Affiliated Drum Tower Hospital, Medical School of Nanjing University, Nanjing, China; ^2^ Department of Cardio-Thoracic Surgery, Nanjing Drum Tower Hospital, Peking Union Medical College & Chinese Academy of Medical Sciences, Graduate School of Peking Union Medical College, Nanjing, China; ^3^ The Jockey Club School of Public Health and Primary Care, The Chinese University of Hong Kong, Hong Kong, Hong Kong SAR, China; ^4^ Department of Pediatric Surgery, Sanya Women and Children’s Hospital, Sanya, China; ^5^ Department of Cardio-Thoracic Surgery, Shanghai Children’s Medical Center, School of Medicine, Shanghai Jiao Tong University, Shanghai, China

**Keywords:** sivelestat, cardiopulmonary bypass, acute lung injury, cardiovascular surgery, outcomes

In the published article, there was an error in **Figures 4B–D** as published. The blue lines were mistakenly identified as the Sivelestat group and the red lines were mistakenly identified as the Control group. The corrected Figure 4 and its caption “**(A)** The oxygen index (PaO_2_/FiO_2_), P<0.01; ※:P< 0.05, †:P> 0.05. **(B)** The procalcitonin, P<0.01; ※:P< 0.05, †:P> 0.05. **(C)** The interleukin-6, P=0.02; ※:P< 0.05, †:P> 0.05. **(D)** The C reactive protein, P<0.01; ※:P< 0.05, †:P> 0.05.” appear below.

**Figure 4:**
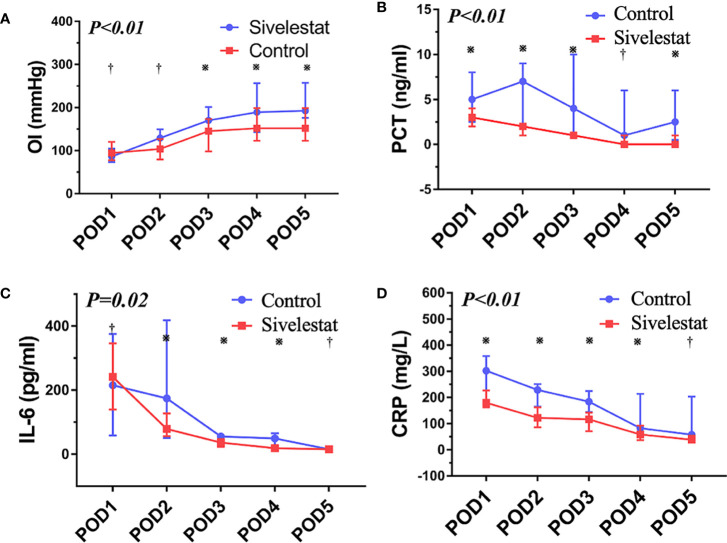
**(A)** The oxygen index (PaO_2_/FiO_2_), P < 0.01; ※:P < 0.05, ^†^:P > 0.05. **(B)** The procalcitonin, P < 0.01; ※:P < 0.05, ^†^:P > 0.05. **(C)** The interleukin-6, P=0.02; ※:P < 0.05, ^†^:P > 0.05. **(D)** The C reactive protein, P < 0.01; ※:P < 0.05, ^†^:P > 0.05.

The authors apologize for this error and state that this does not change the scientific conclusions of the article in any way. The original article has been updated.

