# Supplementary material for: The neutrophil elastase inhibitor, sivelestat, attenuates acute lung injury in patients with cardiopulmonary bypass
Source: Front Immunol. 2023 Jan 24;14:1082830. doi: 10.3389/fimmu.2023.1082830 (PMC9902923; doi:10.3389/fimmu.2023.1082830)

Online supplementary

After intravenous anesthesia induction [sufentanil (0.5-1.0 mg/kg), etomidate (0.1-0.4 mg/kg), vecuronium (0.07-0.15 mg/kg), and midazolam (0.1-0.4 mg/kg)] orotracheal intubation was performed. Anesthesia was maintained with propofol (25-75mg/kg/min), remifentanil (0.1-0.2mg/kg/min), vecuronium (1.0-2.0mg/kg/min), and sevoflurane (<4.0%) in oxygen. Cefuroxime sodium were prophylactically administered before anesthesia. Mechanical ventilation was used before and after CPB. The parameters of ventilation were as follows: positive end-expiratory pressure (PEEP) 4 to 7 cmH_2_O, tidal volume 6 to 10 mL/kg, and a fraction of inspired oxygen (FiO_2_) of 50% to 100%. The insulin infusion was routinely administrated when blood glucose values were greater than 8 to 9 mmol/L (160mg/dL).

The ascending aorta or femoral artery or axillary artery were cannulated with a patient-size-appropriate cannula. Venous cannulations were chosen with separate cannulas in the superior and inferior vena cava. Based on an active clotting time of more than 480s, heparin (200-400 U/kg) was used to achieve anticoagulation. The systemic temperature was kept in a range of 32℃ to 34℃. The DHCA temperature was kept in a range of 22℃ to 24℃. The CPB circuit was primed with 1500-2000 ml of sodium acetate Ringer’s injection, 10-30g of albumin, and 2.5g of magnesium sulfate injection (concentration: 10%). The initial volume of the antegrade cold blood cardioplegia solution (4:1 ratio) was needed for the cessation of all cardiac electrical activity but never less than 20 ml/kg. Cardiac arrest was maintained, after 30 minutes of initiated antegrade infusion, by the retrograde infusion of 10ml/kg of blood cardioplegia solution every 15 minutes. The antegrade strategy would be following implemented if retrograde infusion had been used 3 times. All patients were transferred to the intensive care unit (ICU) after surgery and then were extubated within postoperative 24 hours. Patients in the sivelestat group would be given sivelestat (0.2mg/kg/h) from the first postoperative day (POD1) to the third postoperative day (POD3).

Each patient was transferred to the cardiac ICU after surgery. Blood gas analyses were performed at the time of admission to the ICU, and every 2 hours after surgery. These blood gas analyses were used to adjust mechanical ventilation [(Nellcor Puritan Bennett Ireland, Galway, Ireland) and Dra ̈ger Savina ventilators (Dra ̈gerwerk AG & Co. KGaA, Lu ̈beck, Germany)]. The parameters of mechanical ventilation were as follows: the fraction of inspired O_2_ (FiO_2_: 0.4-0.9), positive end-expiratory pressure (PEEP: 4-10cmH_2_O), target PaO_2_/FiO_2_ >300 mmHg, PaCO_2_(35-45 mm Hg), arterial lactate < 2mmol/L and pH (7.35-7.45). Blood samples for C-reactive protein (CRP), white blood cell (WBC), interleukin-6 (IL-6) and procalcitonin (PCT), etc. measurement and other markers were obtained at admission and then on POD1, POD2, the third postoperative day (POD3), the fourth postoperative day (POD4) and the fifth postoperative day (POD5).

eFig.1: The standardized mean difference


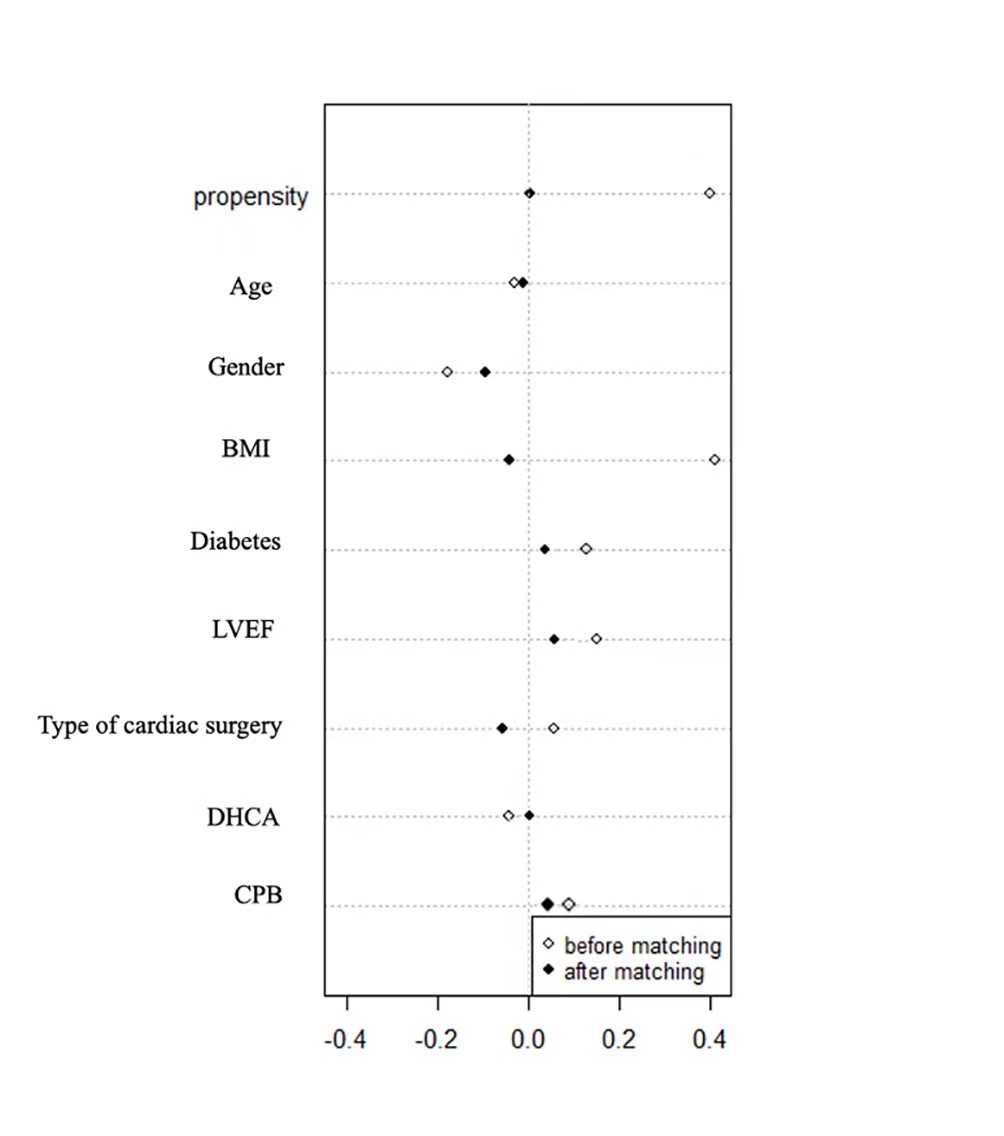

Supplement: Supplementary file 1 [file DataSheet_1.docx]
